# Pharmaceutical Potential of High-Altitude Plants for Fatigue-Related Disorders: A Review

**DOI:** 10.3390/plants11152004

**Published:** 2022-07-31

**Authors:** Hongkang Zhu, Chang Liu, He Qian

**Affiliations:** 1 State Key Laboratory of Food Science and Technology, Jiangnan University, Wuxi 214122, China; 7210112118@stu.jiangnan.edu.cn (H.Z.); 7200112080@stu.jiangnan.edu.cn (C.L.); 2 Collaborative Innovation Center of Food Safety and Quality Control in Jiangsu Province, Jiangnan University, Wuxi 214122, China; 3 School of Food Science and Technology, Jiangnan University, Wuxi 214122, China

**Keywords:** high-altitude plants, natural medicine, anti-fatigue, plateau, disorder, sub-health

## Abstract

Natural plants from plateaus have been the richest source of secondary metabolites extensively used in traditional and modern health care systems. They were submitted to years of natural selection, co-evolved within that habitat, and show significant anti-fatigue-related pharmacological effects. However, currently, no review on high-altitude plants with anti-fatigue related properties has been published yet. This study summarized several Chinese traditional high-altitude plants, including *Rhodiola rosea* L., *Crocus sativus* L., *Lepidium meyenii* W., *Hippophaerhamnoides* L., which are widely used in the Qinghai–Tibet Plateau and surrounding mountains, as well as herbal markets in the plains. Based on phytopharmacology studies, deeper questions can be further revealed regarding how these plants regulate fatigue and related mental or physical disease conditions. Many active derivatives in high-altitude medical plants show therapeutic potential for the management of fatigue and related disorders. Therefore, high-altitude plants significantly relieve central or peripheral fatigue by acting as neuroprotective agents, energy supplements, metabolism regulators, antioxidant, and inflammatory response inhibitors. Their applications on the highland or flatland and prospects in natural medicine are further forecast, which may open treatments to reduce or prevent fatigue-related disorders in populations with sub-optimal health.

## 1. Introduction

In recent years, the challenges of sub-health, aging of the rising population, as well as the prevalence of chronic diseases need perfect healthcare systems to support human fitness. Meanwhile, major life stressors are among the strongest proximal risk factors for fatigue in the pathological or sub-healthy state. Actually, numerous traditional Chinese medicines (TCM) have been shown to exert significant anti-fatigue actions, mainly through regulating the central nervous system, supplementing vital energy, and boosting muscle contractility, which could provide additional natural compounds for management of fatigue [1]. As for regulating physical fatigue, the multiple pharmacological activities of alpine plants (especially Tibetan medicines) have been demonstrated, such as for *Rhodiola rosea* L. [2], *Crocus sativus* L. [3], *Lepidium meyenii* (Walp.) [4], *Hippophaerhamnoides* L. [5], etc. Notably, these plants have also been widely used in the prevention and treatment of fatigue-related symptoms (i.e., weakness, bradykinesia, depressed mood) for a long time. The mechanism underlying their effects remains largely unclear, but the provision of these supplements has yielded improvement in aerobic performance and not only in high-altitude hypoxia environments [6].

**Figure 1:**
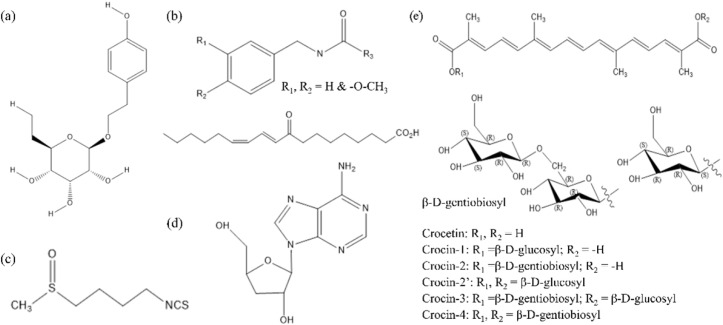
Structures of some special functional ingredients from high-altitude plants with anti-fatigue effect. (**a**) Salidroside from *Rhodiola rosea* L., (**b**) macaenes and macamides from *Lepidium meyenii* W., (**c**) sulforaphane from cruciferous family (*Brassica rapa* L.), (**d**) cordycepin from *Cordyceps sinensis Sacc.*, (**e**) crocin from *Crocus sativus* L.

There is much evidence suggesting that the growth and development of plants influence the production of secondary metabolites [7], and the highlighted medicinal value in a multitude of alpine plants might be related to thousands of years of adaptation and evolution in the high-altitude mountain ecosystem. Pharmacological studies have proved that various active ingredients (i.e., polysaccharide, flavonoids, triterpenes, and alkaloids) in Chinese medicinal plants might improve physical resistance to fatigue [8]: polysaccharides could promote energy supply (glycogen synthesis and metabolism); flavonoids could enhance antioxidant capacity; triterpenes and alkaloids could increase the reservation of glycogen substances and reduce the accumulation of metabolites. Notably, some special functional ingredients are involved in managing the crucial process of fatigue, such as salidroside [9], macaenes and macamides [10], sulforaphane [11], cordycepin [12], crocin [13], etc. (Figure 1). Thus, high-altitude medicinal plants and fruits are receiving increasing interest for their versatile pharmacological and biological activities [7].

**Figure 2:**
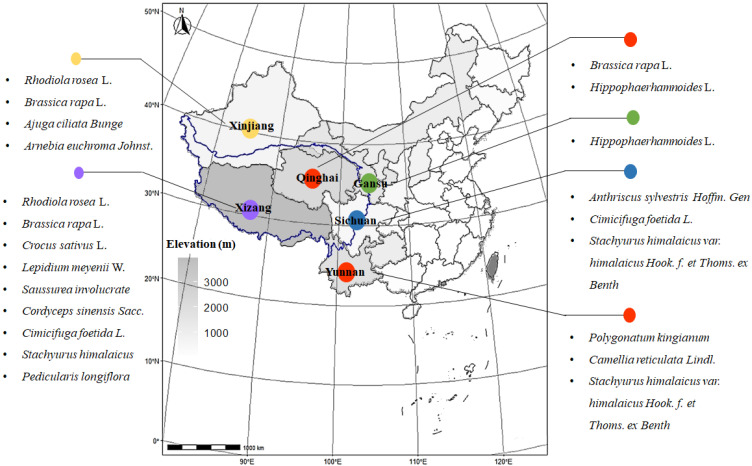
Distribution of high-altitude plants with anti-fatigue effect. Here, 15 representative high-altitude plants with anti-fatigue effect are summarized, which are distributed among the Tibetan Plateau and surrounding mountains (mainly across six provinces). The boundary of the Qinghai–Tibet Plateau in the map is circled in blue (solid line). Color represents average elevation; the darker the color, the higher the area.

Much has been said recently about the use of Chinese traditional medicinal plants in anti-fatigue, especially about those cultivated at high altitude. In this review, we investigated Zhonghua Bencao, Flora of China, Standard of Tibetan Medicines in Sichuan Province (2020) and scientific databases, and then summarized 15 representative Chinese traditional high-altitude plants with corroborating anti-fatigue efficacy (Figure 2). Most of them are distributed among the Tibetan Plateau and surrounding mountains, as well as other regions such as Yungui Plateau and Junín plateau (Peru). The detailed Latin name, family, elevation, distributions, and main active ingredients were supplemented in Table 1. Based on plant physiology and pharmacology, a biologically plausible, multi-level theory was proposed that describes plant pharmacology mechanisms that link medical plant adaptation to harsh environmental stress with human internal biological processes to alleviate physical fatigue. Central to this intersectional adaptation theory is the hypothesis that some functional components in high-altitude plants share similar routes of delivery and modes of action in the management of physical fatigue. Based on plant pharmacology, deeper questions can be revealed regarding how alpine plants regulate symptoms of fatigue and relationships to mental or physical disease conditions. This work may also suggest new opportunities for preventing and managing fatigue with high-altitude plants via multi-targets (i.e., neuroprotection, metabolism regulation, anti-oxidation, or anti-inflammation) and new directions.

## 2. Habitat and Adaptation

Exposure to various natural environmental factors leads to subject stress in medical plants, which may in turn affect multiple biological processes [14]. Plateau environmental stress on medical plants during their evolution has attracted considerable attention. Along with increase in altitude, the environment becomes harsher, which results in nutritional deficiencies, frost, ultraviolet radiation, and oxidative stress. Plant-related metabolites are highly abundant “background” metabolites that are involved with plant physiology changes, showing differential abundance under various stress conditions. There is much evidence suggesting that growth and development of plants influence the production of secondary metabolites [7], and the highlighted medicinal values in a multitude of alpine plants might benefit from thousands of years of adaptation and evolution in high-altitude mountain ecosystems. Shi et al. [15] observed that immune genes in Maca root were up-regulated during daytime and stress tolerance genes were up-regulated from October to December in the Yungui Plateau (3300 m above sea level). Alternative splicing (AS), coupled to nonsense-mediated decay (NMD), might act as an essential mechanism for Maca in its adaptation to a high-altitude ecosystem. These plants have to coordinate remobilization and relocation of metabolites in an extreme climate with low oxygen concentration and strong ultraviolet radiation in the Junín plateau (4138 m above sea level) [16]. Supplements of high-altitude medicines or natural products containing characteristic ingredients are associated with several other nutrients, so they seem to present ergogenic effects. Generally, soluble sugars, fatty acids, and alkaloids in alpine plants are highly sensitive to environmental stress. Nonetheless, it is not possible to attribute anti-fatigue properties to these ingredients only. Recent technological developments have revealed adaptive mechanisms of medical plants along the altitude gradient at the level of proteomics [17] and metabolomics [18]. Thus, high-altitude medicinal plants and fruits have attracted increasing interest for their versatile pharmacological and biological activities [7].

## 3. Environmental Stresses of Natural Plants at High Altitude

### 3.1. Low Oxygen Concentration

Medicinal plants have been the richest source of secondary metabolites extensively used in traditional and modern healthcare systems. The extreme environmental conditions of high-altitude region (i.e., low oxygen concentration, high ultraviolet (UV) radiation, extreme temperature, salinity, etc.) might affect plant growth and distribution [19]. Yet, how medicinal plants in response to high-altitude environmental stresses is not sufficiently studied [17]. In total, about 90 species of *Crassulaceae* family are native to the arctic regions of Eurasia and North America, which are cold-tolerant and characterized by hypoxia and strong UV radiation [20]. Compared with lowland cultivation, higher-altitude colonized plants are exposed to rougher conditions. Their vegetation periods are shorter and produce more rhizome than root biomass, with higher content of bio-active compositions such as salidroside, tyrosol, rosarin, rosavin, and cinnamyl alcohol (trans-cinnamic alcohol) [21].

### 3.2. Ultraviolet Radiation

High-altitude plants might remobilize and relocate some metabolites between source and sink organs [16]. In below-ground organs, the more bio-active ingredient of carbohydrate, glucosinolates, phenolics compounds, etc. were synthesized by some possible signaling pathways, which were reactive oxygen species (ROS)-related and/or UV-specific photoreceptors. Docking and enzyme kinetic studies indicated that enzymes of flavonoid biosynthesis pathway might confer plants with tolerance to UV-B and dehydration in planta [22]. Therefore, plants activated UV-B-induced compounds, such as flavonoids, antioxidants, etc., to protect the photosynthetic apparatus from permanent damage [23].

### 3.3. Extreme Climates

The extreme climate of high-altitude regions, defined by stressors such as low temperature, limits plant growth and distribution, which affects the life cycle of plants [17]. *Saussurea involucrata* (Kar. et Kir.), a rare traditional medicinal plant, grows in high mountains covered by snow in the Tibet and Tianshan Mountains areas of China [24]. It takes more than 8 years to mature before harvest, under rather harsh climatic conditions. Different from the alpine plants above, *Saussurea involucrata* might be more involved in chilling and freezing tolerance via the cold-response signaling pathways and molecular metabolic reactions [25].

### 3.4. Other Factors

The plateau ecosystem is recognized as the most vulnerable to various factors, such as natural ecological elements (symbiotic microbial community, herbivores) and anthropogenic activities (metal toxicity, air pollution). An analysis [26] of days to flowering (DTF) on *Brassica* species (Qinghai–Tibet Plateau) showed that the external environment affected herbivore pressure, vegetative growth, and its genetic self-regulation. In addition, the genome size (GS) of turnip from plateau environments (Qinghai–Tibetan Plateau) was always smaller than that from lower latitudes (Yunnan Plateau), where 15.5% variation was observed [27]. Thus, turnip was conventionally named with regional characteristics for distinguishment (e.g., ‘Tibetan turnip’) and became a main source of food for inhabitants. Compared with plain areas, higher regions with arid and stressful environments (e.g., Qinghai–Tibet Plateau and Xinjiang areas) have proven profitable for the accumulation of the total glucosinolate in *Brassica rapa* L. [28,29].

## 4. Pharmacological Effects on Treatment of Fatigue-Related Disorders

High-altitude plants contain flavonoids, polysaccharides, phenols, triterpenes, alkaloids, glycosides, and other main active ingredients (Table 1), and their pharmacological effects of anti-fatigue are mainly concentrated in scavenging free radicals, antioxidants, anti-inflammatory, and other effects such as neuromodulation and immune stimulation (Table S1).

### 4.1. Neuroprotective Agent: Adjustment of the Level of the Central Neurotransmitters

It is generally believed that central serotonergic and dopaminergic systems are fully engaged in central fatigue and onset of exercise-induced fatigue [30]. Neurotransmitter receptors, along with their transporters, are thought to be very important markers in the fatigue process. The enhancement of brain dopamine (DA), noradrenaline (NA), neural activity, and inhibition of the synthesis and metabolism of 5-HT could postpone the occurrence of fatigue [31]. The positive effects of alpine plants on exercise capacity declination via neuroprotection/stimulation during long-time exercise has been observed. Macamides demonstrated similar medicinal properties to cannabinoids via CB_1_ receptor activation in the central nervous system [32]. *Rhodiola* and salidroside are also well-known for their neuroprotective and antidepressant activity [33]. Of course, alpine plants with compatibility and multiple targets acted on not only the autonomic nervous system (ANS) but also the hypothalamic pituitary–adrenal axis (HPA) to protect the central nervous system and resist fatigue [34]. The HPA axis is an important regulator of neurotransmitters, metabolites, and inflammatory cytokines. Sea buckthorn suppressed cortisol, adreno-cortico-tropic-hormone (ACTH) levels, and increased DA and norepinephrine levels [35].

### 4.2. Energy Supply and Metabolism: Maintainance of Energy Homeostasis

Physiological fatigue can also be defined as a reduction in the force output and energy-generating capacity of a body after chronic exposure to work or usual activities at the same intensity. When exercise leads to exhaustion, peripheral fatigue and activation of muscle afferents probably contribute to limiting exercise performance [36]. Therefore, energy homeostasis maintains exercise capacity of the body, preventing exhaustion of physical energy reserves such as adenosine triphosphate (ATP), glycogen, and fat. Most traditional medicinal plants, especially those with polysaccharides such as are found in Tibetan medicines, generally possess the capacity to improve glycogen stores by increasing glycogen storage or delaying glycogen consumption, or both [5,8]. For example, ethanol extract of Maca increased glycogen uptake in an adipocyte cell line by mediating phosphorylation of insulin receptor (IR) and phosphatidylinositol-3-kinase/protein kinase B (PI3K/AKT) pathways [37]. Generally, anti-fatigue natural plants improve exercise mainly by increasing glycogen storage, but also by regulating related metabolism. Various studies have demonstrated that supplements of high-altitude plants or active ingredients promote the recovery of fatigue in mice by regulating glucose metabolism [38], lipid metabolism [39], and energy metabolism [40]. The activation of amp-dependent protein kinase (AMPK) is the axis of energy homeostasis, highly involved in the regulation of biological energy metabolism. Some alpine plants [41] triggered AMPK catabolic pathways that produced ATP, while inhibiting energy-consuming anabolic activities mediated by mTOR, such as *Chikusetsu saponin* Iva (1400–4000 m) [42]. PPARγ and its coactivator-1α (PGC-1α), downstream genes of AMPK, were shown to have effects on glucose metabolism and energy metabolism-related genes [43], which were stimulated by *Rhodiola sacra* [41], Maca [44], or *Saussurea involucrata* (rutin) [45]. Recently, increasing evidence suggests that mitochondria are essential for maintaining energy homeostasis. Extract of Maca (macamides) [37], *Rhodiola sacra* (salidroside) [46], possessed marked anti-fatigue effects, which might enhance mitochondrial quality control, including mitophagy, mitochondrial dynamics, and biogenesis in mice.

### 4.3. Removal of Accumulated Metabolites: Enhancement of Muscle and Organ Adaptation

Metabolic stress, a physiological process during exercise, accelerates the declination of exercise capacity in muscle [47]. Blood lactate (BLA), blood urea nitrogen (BUN), and lactic dehydrogenase (LDH) activity are important indicators of body fatigue. They interfere with non-oxidative ATP production and glycogenolysis, protein and amino acid metabolism, ATP generation capacity, and removal of lactic acid in skeletal muscle. High-altitude plants have functions of resisting fatigue, where they reduce metabolites accumulation and thus slow down exhaustion time and improve exercise endurance. The extract of *Rhodiola rosea* [40], Maca [4], sea buckthorn [48], and other anti-fatigue alpine plants [49] could clear the accumulation of BLA and BUN. Zhang et al. [10] found that macamides could increase LDH and creatine kinase (CK) levels, effectively eliminating BLA and BUN to attenuate skeletal muscle and myocardium damage. During high-intensity exercises, other metabolites such as malondialdehyde (MDA), lactate, phosphate inorganic (Pi), and ions of hydrogen (H^+^) are also produced to damage the muscle, causing the dissolution of muscle cells. Supplementation of polysaccharides [50], and some flavonoids [45] and alkaloids [51], significantly reduced CK levels and enhanced exercise endurance in mice. Additionally, elevation of alanine aminotransferase (ALT) and aspartate aminotransferase (AST), glutamic oxaloacetic transaminase (GOT), and glutamic pyruvic transaminase (GPT) are predictors of heart and hepatocyte injury induced by intense physical activity. These increasing cytosolic enzyme level indicate that dysfunction of organs has occurred or is occurring. Chronic *Rhodiola rosea* extract supplementation significantly enhanced content of liver glycogen, and reduced GOT and GPT levels in a dose-dependent manner [40]. Thus, exercise-induced metabolic stress is alleviated by anti-fatigue plants via multiple metabolic networks that remove metabolite accumulation in a series of metabolic pathways, consistent with “catastrophe” models of fatigue [52].

### 4.4. Free Radical Scavenger: Antioxidant Activity

Free radicals, such as hydroxyl radicals and superoxide anion radicals, are some by-products produced in the process of metabolism of organisms. The generation and removal are in a dynamic equilibrium under normal circumstance. However, when exercising too vigorously, the acutely generated ROS are out of balance. Thus, free radicals accumulate and lead to oxidative stress, which is partly regarded as a causal factor for muscle damage and body tiredness [53]. Thus, exogenous sources of antioxidants are vital to cope with oxidative stress-induced fatigue in organisms, apart from endogenous antioxidant defense mechanisms [54]. Supplements of exogenous sources of antioxidants from high-altitude plants have a positive significance in the repair of oxidative injuries. In this way, physiological fatigue is eliminated via moving ROS, relieving oxidative stress, and keeping the balance between ROS and antioxidant system. A large number of high-altitude medicines [46,55,56] prolonged mice exercise endurance via enzymatic antioxidant system, including superoxide dismutase (SOD), glutathione peroxidase (GSH-Px), and catalase (CAT). Extracts of Maca could activate the phosphorylation of AMPK, which was an important target for energy metabolism and treatment of fatigue [37]. In addition, Nrf2 is a new cell antioxidant regulator, while sulforaphane can induce expression [38,57]. Salidroside inhibited oxidative stress and inflammation by inducing Nox2 and Nox4 and reducing Nrf2 and NQO1 in denervated muscles [58].

### 4.5. Inflammatory Response Inhibitor: Anti-Inflammatory Activity

Exhaustive exercise leads to excessive ROS production and accumulation and causes rapid release of pro-inflammatory cytokines, such as tumor necrosis factor-α (TNF-α), interleukin-1β (IL-1β), and interleukin-6 (IL-6), damaging biomembranes, proteins, and DNA. It is generally believed to be a convincing contributor to bodily dysfunctions, such as inflammatory diseases, vascular disorder, cognitive impairment, aging, and chronic fatigue [59]. High-altitude plants with anti-inflammatory activities, such as ethanoic extract from Maca [60], significantly inhibited the activities of TNF-α, IL-6, and IL-1β, and alleviated exercise-induced fatigue. In addition, macamides could also interacted the expression of analog of exogenous anandamide (AEA) and receptor (CB_1_) [32], so that it might reduce exercise-induced inflammatory nociception mediated by endocannabinoid [61,62]. IL-10 is an anti-inflammatory cytokine that can reduce antigen presentation, negatively regulating the activity of pro-inflammatory cytokines. For example, *Rhodiola rosea* [63] significantly potentiated serum levels of IL-10, thus preventing attenuation of pro-inflammatory cytokines as well as chemcytokines release. Furthermore, herbal supplements like sea buckthorn [64] downregulated the master immune transcription factor nuclear factor kappa B (NF-κB). Hou et al. [35] also found that sea buckthorn significantly inhibited the increase of serum corticosterone and adrenaline levels through the HPA axis under chronic stress (exhausted swimming, 10 min/day, 21 days). Meanwhile, mitogen-activated protein kinases (MAPK) are another important factor in regulation of inflammation. Anti-inflammatory activity of salidroside was partly linked to the blocking of the both NF-κB and MAPK signaling pathways [65].

## 5. Conclusions and Prospects

Plateau environmental stress on natural plants during their evolution has attracted considerable attention [66]. Along with an increase in altitude, the environment becomes harsher, which results in nutritional deficiencies, frost, ultraviolet radiation, and oxidative stress. Plant-related metabolites are highly abundant “background” metabolites that are involved with plant physiology changes, showing differential abundance under various stress conditions. Generally, soluble sugars, fatty acids, and alkaloids in alpine plants are highly sensitive to environmental stress. Nonetheless, it is not possible to attribute anti-fatigue properties to these ingredients only. Alpine plants tend to be potential therapeutic strategies for greater tolerance to fatigue in traditional usage. The traditional medicinal high-altitude plants are also known as “Daodi herb” in TCM, which refers to geo-authentic/authentic/genuine or superior medicinal herbal material. There is growing evidence that these plants may be also an advantageous strategy for the treatment of fatigue or sub-health, mainly through the aspects of neuroprotection and regulation of neurotransmitter disorder, regulation of energy supply and metabolism, delaying the accumulation of metabolites and promotion of mitochondrial function, antioxidant stress, and inflammatory response inhibition. Thus, the anti-fatigue ability of these plants and nutraceuticals might be highly correlated with stress acclimation.

In recent years, there are several TCM plant databases, such as TCM Systems Pharmacology Database (TCMSP), and TCM Integrated Database (TCMID), that enhance and expand the medicinal applications of plants in many ways. However, they are not specifically designed for alpine plants and may lead to the absence of alpine plants. Thus, a regional database with alpine plants is needed in the future, and all of them for any pharmacological activity potentially useful against fatigue and related disorders. However, much still remains to be done since an alpine plant database would be made up of hundreds of unexploited medicinal plants. We expect that this review will provide a scientific basis for understanding medicinal plants with anti-fatigue effects at high altitude. These natural plants, products, or prescriptions deserve to be further clarified for market positioning, which is critical for relieving the stress of fatigue and improving quality of life and well-being in specific sub-health groups living on either plain or plateau.

## Figures and Tables

**Table 1:** The anti-fatigue effects and main active ingredients from high-altitude plants.

No.	Latin Name	Family	Elevation/m	Distributions	Used Part	Main Active Ingredients
1	*Rhodiola rosea* L.	*Crassulaceae*	2800–	Tibet, Xinjiang	Root, rhizome	Flavonoids, salidroside
2	*Brassica rapa* L.	*Brassica*	3500-	Tibet, Xinjiang, Qinghai	Root	Polysaccharide, isothiocyanates
3	*Crocus sativus* L.	*Iridaceae*	5000-	Tibet	Filament	Flavonoids, crocin
4	*Lepidium meyenii* W.	*Brassicaceae*	3800-	Tibet	Root	Polysaccharide, alkaloids (macamides)
5	*Hippophaerhamnoides* L.	*Elaeagnaceae*	800–	Qinghai, Gansu	Fruit	Flavonoids
6	*Saussurea involucrata Sch.-Bip.*	*Compositae*	4300-	Tibet	Flower	Flavonoids
7	*Cordyceps sinensis Sacc.*	*Clavicipitaceae*	5000-	Tibet	Complex	Polysaccharide, cordycepin
8	*Ajuga ciliata Bunge*	*Labiatae*	2500–	Xinjiang	Whole grass	Flavonoids, triterpenes
9	*Arnebia euchroma Johnst.*	*Boraginaceae*	2500–	Xinjiang	Root	Polysaccharide
10	*Anthriscus sylvestris Hoffm. Gen*	*Umbelliferae*	4500-	Liaoning, Sichuan	Root	Lactones
11	*Polygonatum kingianum*	*Liliaceae*	700–	Yunnan	Root	Polysaccharide, flavonoids, triterpenes
12	*Cimicifuga foetida L.*	*Ranunculaceae*	1700–	Tibet, Liaoning, Sichuan	Root	Triterpenes
13	*Stachyurus himalaicus var. himalaicus Hook. f. et Thoms. ex Benth*	*Stachyuraceae*	1500–	Tibet, Yunnan, Sichuan	Stem pith	Polyphenols, triterpenes
14	*Camellia reticulata Lindl.*	*Theaceae*	2200–	Yunnan	Flower, leaves	Polyphenols
15	*Pedicularis longiflora var. tubiformis*	*Pedicularis*	2700–	Tibet	Whole grass	Flavonoids, boschnaloside

## Data Availability

Not applicable.

## Supplementary Materials

The following supporting information can be downloaded at: https://www.mdpi.com/article/10.3390/plants11152004/s1, Table S1: The main active ingredients and anti-fatigue mechanism of high-altitude plants. References [67,68,69,70,71,72,73,74,75,76,77,78,79,80,81,82,83,84,85,86,87,88,89,90,91,92,93,94,95,96,97,98] are cited in the supplementary materials.

## Abbreviations

AEA, analog of exogenous anandamide; ALT, alanine aminotransferase; AMPK, amp-dependent protein kinase; ANS, autonomic nervous system; AS, Alternative splicing; AST, aspartate aminotransferase; ATCH, adreno-cortico-tropic-hormone; BLA, blood lactate; BUN blood urea nitrogen CAT, catalase; CK, creatine kinase; DA, dopamine; DTF, days to flowering; GOT, glutamic oxaloacetic transaminase; GPT, glutamic pyruvic transaminase; GS, genome size; GSH-Px, glutathione peroxidase; HPA, hypothalamic pituitary-adrenal axis; IL-1β, Interleukin-1β; IL-6, Interleukin-6; IR, insulin receptor; LDH, lactic dehydrogenase; MAPK, mitogen activated protein kinases; NA, noradrenaline; NF-κB, nuclear factor kappa B; NMD, nonsense-mediated decay; PI3K/AKT, phosphatidylinositol-3-kinase/protein kinase B; ROS, reactive oxygen species; SOD, superoxide dismutase; TCM, traditional Chinese medicine; TNF-α, tumor necrosis factor-α; UV, ultraviolet.
