# Supplementary material for: Pharmaceutical Potential of High-Altitude Plants for Fatigue-Related Disorders: A Review
Source: Plants (Basel). 2022 Jul 31;11(15):2004. doi: 10.3390/plants11152004 (PMC9370126; doi:10.3390/plants11152004)
Supplement: Supplementary file 1 [file plants-11-02004-s001.zip › plants-1833851-supplementary.pdf]

plants

MDPI

Supplementary Materials

Table S1. The main active ingredients and anti-fatigue mechanism of high-altitude plants.

No.	Latin Name	Main Active Ingredients	Traditional Use	Mechanism of Anti-Fatigue Activity
1	*Rhodiola rosea* L.	Salidroside [9]	Alleviate fatigue, depression, and cognitive dysfunction [67]	Neuroprotection [67,68], scavenging free radicals, antioxidation [69]
2	*Brassica rapa* L.	Polysaccharides [70]	Tonic and anti-hypoxia, heat-clearing and detoxification, alleviating fatigue [50]	Removal of accumulated metabolites [71], antioxidation [55]
3	*Crocus sativus* L.	Cordycepin [12]	Neurodegenration or metabolic diseases [72]	Anti-inflammatory and antioxidation [10]
4	*Lepidium meyenii* W.	Polysaccharides [4], macaenes, macamides [10]	Increase fertility [73]	Removal of accumulated metabolites, improvement of energy metabolism [74]
5	*Hippophaerhamnoides* L.	Polysaccharides [5], flavonol glycosides [75]	Alleviate fatigue caused by oxygen deficiency [5]	Antioxidation [76]
6	*Saussurea involucrata Sch.-Bip.*	Acacetin, hispidulin, and rutin [77,45]	Alleviate pain and inflammatory [78]	Scavenge free radicals [79], antioxidation [80]
7	*Cordyceps sinensis Sacc.*	Polysaccharides [81], crocin [13],	Prevention of anemia, physical weakness	Increase storage of energy, antioxidation [82]
8	*Ajuga ciliata Bunge*	Flavones, neo-Clerodane diterpenes [83]	Swelling, rheumatic pain, and traumatic injury	Anti-inflammatory [84]
9	*Arnebia euchroma Johnst.*	Shikonin, polysaccharide	Antitumor, immunomodulatory [85]	Scavenge free radicals [86], antioxidation [87]
10	*Anthriscus sylvestris Hoffm. Gen*	Lignan	Antitumor, anti-inflammatory [88]	Anti-inflammatory [88]
11	*Polygonatum kingianum*	Saponins, polysaccharides and lectins [89]	Diabetes, fatigue, feebleness, indigestion [89].	Promote energy metabolism and antioxidation ability [90]
12	*Cimicifuga foetida L.*	Triterpenoids	Perimenopausal symptoms [91]	Neuroprotection [91]
13	*Stachyurus himalaicus var. himalaicus Hook. f. et Thoms. ex Benth*	Triterpenoids [92]	Galactopoietic, diuretic, dropsy and gonorrhea [93]	Antioxidation and anti-inflammatory
14	*Camellia reticulata Lindl.*	Flavonoid glycosides [94], anthocyanins [95]	Heat-clearing, damp-draining, blood-cooling, and detoxifying	Scavenge free radicals [96]
15	*Pedicularis longiflora var. tubiformis*	Saponins	Leucorrhoea, fevers, sterility, rheumatism, general debility, collapse, urinary problems [97]	Antioxidation [98]

References follow the main text
